# Physical Association of PDK1 with AKT1 Is Sufficient for Pathway Activation Independent of Membrane Localization and Phosphatidylinositol 3 Kinase

**DOI:** 10.1371/journal.pone.0009910

**Published:** 2010-03-26

**Authors:** Zhiyong Ding, Jiyong Liang, Jin Li, Yiling Lu, Vathsala Ariyaratna, Zhimin Lu, Michael A. Davies, John K. Westwick, Gordon B. Mills

**Affiliations:** 1 Department of Systems Biology, The University of Texas M.D. Anderson Cancer Center, Houston, Texas, United States of America; 2 Department of Neuro-Oncology, The University of Texas M.D. Anderson Cancer Center, Houston, Texas, United States of America; 3 Department of Melanoma Medical Oncology, The University of Texas M.D. Anderson Cancer Center, Houston, Texas, United States of America; 4 Odyssey Thera, Inc., San Ramon, California, United States of America; Roswell Park Cancer Institute, United States of America

## Abstract

Frequent activation of the AKT serine-threonine kinase in cancer confers resistance to therapy. AKT is activated by a multi-step process involving phosphatidylinositide (PtdIns) phosphate-mediated recruitment of AKT and its upstream kinases, including 3-Phosphoinositide-dependent kinase 1 (PDK1), to the inner surface of the cell membrane. PDK1 in the appropriate context phosphorylates AKT at threonine 308 (T308) to activate AKT. Whether PtdIns(3,4,5)Ps (PtdInsP3) binding and AKT membrane translocation mediate functions other than formation of a functional PDK1::AKT complex have not been fully elucidated. We fused complementary fragments of intensely fluorescent protein (IFP) to AKT1 and PDK1 to induce a stable complex to study the prerequisites of AKT1 phosphorylation and function. In the stabilized PDK1-IFPC::IFPN-AKT1 complex, AKT1 T308 phosphorylation was independent of PtdIns, as demonstrated by treatment with Phosphatidylinositol 3 Kinase (PI3K) inhibitors. Further when interaction with PtdIns and the cell membrane was prevented by creating PH-domain mutants of AKT1 (R25A) and PDK1 (R474A), AKT1 phosphorylation on T308 was maintained in the PDK1-IFPC::IFPN-AKT1 complex. The PDK1-IFPC::IFPN-AKT1 complex was sufficient for phosphorylation of known AKT substrates, and conferred resistance to inhibitors of PI3K (LY294002, PI103, GDC0941 and TGX286) but not inhibitors of the downstream TORC1 complex (rapamycin). Thus the locus of action of targeted therapeutics can be elucidated by the constitutively active AKT1 complex. Our data indicate that PtdIns and membrane localization are not required for AKT phosphorylation and activation, but rather serve to induce a functional physical interaction between PDK1 and AKT. The PDK1-IFPC::IFPN-AKT1 complex provides a cell-based platform to examine specificity of drugs targeting PI3K pathway components.

## Introduction

The serine/threonine kinase AKT (also known as protein kinase B, PKB), comprising a group of 3 isoforms, AKT1, AKT2, and AKT3, plays a central role in cell metabolism, survival, growth, motility, and tumorigenesis [1], [2]. AKT is frequently activated in cancer by amplification of growth factor receptors (HER2/neu, EGFR), activating mutations of intracellular kinases (PIK3CA), amplification or mutation of AKT isoforms, and inactivation of phosphatases (PTEN) [3]. The development of effective, non-toxic inhibitors that target AKT activation is thus an active field of investigation. AKT is activated by a cascade of events that is initiated by the recruitment of class I PI3Ks to the cell membrane, as occurs following activation of transmembrane receptor tyrosine kinases. Class IA PI3K phosphorylates PtdIns(4,5)P (PtdInsP2) to form PtdIns(3,4,5)Ps (PtdInsP3) on the inner cell membrane, which recruits proteins with pleckstrin homology (PH) domains including AKT and PDK1 to the cell membrane. Upon recruitment to the cell membrane AKT is phosphorylated at two critical residues, T308 in the activation T loop, and S473 in the hydrophobic domain, to fully activate its kinase activity. PDK1 [4] phosphorylates AKT at T308, and mTORC2 [5] as well as other potential PDK2s phosphorylates AKT at S473. The phosphorylated, active AKT then translocates from the cell membrane to other cell compartments to phosphorylate its downstream substrates that critically regulate many cellular processes [6]. Recent studies have uncovered many details in each step of the activation process. AKT has been shown to form complex with PDK1 in both resting and stimulated cells[7], [8]. A “PH-in” conformation of AKT prevents PDK1 from phosphorylating AKT in resting cells. Association of AKT and potentially PDK1 with PtdIns alters the PH-in conformation allowing phosphorylation of AKT at T308[7], [8]. Multiple scaffold proteins, including PAK and Freud-1, have also been identified to facilitate AKT association with PDK1 promoting AKT translocation and phosphorylation[9], [10]. Ubiquitination has also been shown to promote AKT translocation and activation[11]. Therefore, AKT activation is an exquisitely regulated and context-dependent process. However, many of the prerequisites to AKT phosphorylation have not been fully clarified, i.e., whether PtdInsP3 binding or membrane localization is indispensable for AKT phosphorylation by PDK1. An improved understanding of this requirement for AKT activation could help refine drug development approaches targeting the activated AKT pathway.

We hypothesized that PtdInsP3 binding and membrane localization is not required for AKT phosphorylation and activation by PDK1 if the two proteins could be brought into proximity as stable complex with proper conformation by alternative mechanisms. Previously, we have used protein-fragment complementation assays (PCA) to screen for protein-protein interactions with AKT1 in intact cells [12]. In PCA, a reporter protein such as a monomeric enzyme or a fluorescent protein (GFP or a variant thereof, IFP will be used as an example here) is rationally dissected into 2 fragments that do not reconstitute spontaneously [13], [14], [15]. When each fragment of IFP is fused to one of a pair of interacting protein partners, the subsequent protein interaction places the IFP fragments in proximity restoring IFP molecules and fluorescence. The reconstituted barrel structure of IFP is relatively stable [16] so PCA has the potential to stabilize transient interactions, such as enzyme–substrate interactions. We took advantage of the reconstitution of IFP fragments to generate stable complexes of PDK1 and AKT1 in the absence of PI3K activity and membrane localization. The results show that the physical association of PDK1 with AKT1 induced by reconstituted IFP is sufficient to produce T308 phosphorylation and activation of AKT1 independent of PI3K activation or membrane localization. The constitutively activated PDK1::AKT1 complex provides a cell-based platform to examine drug specificities in the PI3K pathway. The results also demonstrate that the activation of AKT1, and induction of resistance to PI3K pathway inhibitors as well as paclitaxel, can be achieved in the absence of membrane localization of AKT1.

## Results and Discussion

### AKT1 interaction with PDK1 is stabilized by reconstituted IFP

**Figure 1:**
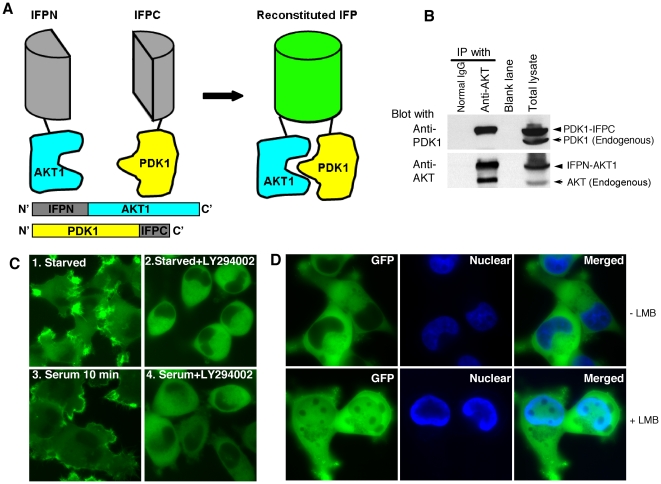
AKT1 interaction with PDK1 stabilized by the reconstituted IFP. (A) Schematic diagram showing the split IFP reconstitution mediated by the interaction between AKT1 and PDK1. IFPN was fused to the N-terminus of AKT1. IFPC was fused to the C-terminus of PDK1. Neither fusion protein was fluorescent. The interaction between AKT1 and PDK1 brings IFPN and IFPC in proximity and reconstitutes IFP fluorescence. The reconstituted IFP stabilizes the PDK1-IFPC::IFPN-AKT1 complex. (B) Association of PDK1-IFPC with IFPN-AKT1. HeLa cells stably expressing the PDK1-IFPC::IFPN-AKT1 complex were lysed in NP40 lysis buffer. Immunoprecipitation was performed with the indicated antibodies (top labels), followed by western blotting with anti-PDK1 (upper blot) and anti-AKT (lower blot). Lane 1, IP with normal IgG. Lane 2, IP with anti-AKT1. Lane 3, blank. Lane 4, total cell lysate. (C) Translocation of the PDK1-IFPC::IFPN-AKT1 complex upon PI3K signaling activation or inhibition. Cells stably expressing the PDK1-IFPC::IFPN-AKT1 complex were serum starved for overnight then treated with LY294002 (20 µM) for 3 hours (image 2), 10% serum for 10 minutes (image 3), and LY294002 (20 µM) for 3 hours and then 10% serum for 10 minutes (image 4). At least 100 cells were examined from different fields for each sample with 100% of the examined cells in each sample showing the same fluorescence localization presented. (D) Accumulation of the PDK1-IFPC::IFPN-AKT1 complex in nucleus with LMB treatment. HeLa cells stably expressing the PDK1-IFPC::IFPN-AKT1 complex were starved overnight and then treated with LMB at 50 nM for 3 hours. Nuclei were stained with Hoechst 33342.

To determine the effect of the interaction of AKT with PDK1 on AKT phosphorylation and activation, we employed PCA technology to create a stable complex containing the two proteins. IFP, a GFP derivative, also known as Venus [17], was used. An IFP N-terminal portion of amino acid 1-158 (IFPN) and an IFP C-terminal portion of amino acid 159-239 (IFPC) were synthesized previously [15]. Neither IFP fragment is fluorescent by itself, nor do they associate spontaneously [15], [18]. IFPN was fused to the N-terminus of AKT1, and IFPC to the C-terminus of PDK1 (Fig. 1A). Following transfection into HeLa cells, PDK1-IFPC was readily immunoprecipitated by anti-AKT antibody and detected with anti-PDK1 antibody. In contrast, endogenous PDK1 was not detected under these conditions suggesting that the endogenous PDK1::AKT interaction was not stable to the immunoprecipitation approach used in this study (Fig. 1B). The results confirmed the physical interaction between PDK1-IFPC and IFPN-AKT1 and stabilization of the complex by the reconstituted IFP. The co-expression of PDK1-IFPC and IFPN-AKT1 resulted in fluorescence (Fig. 1C), indicating that the interaction between AKT1 and PDK1 brought the two IFP fragments into proximity and restored IFP fluorescence, as observed previously [12]. We previously showed that actinin 4 (ACTN4) is an interaction partner of AKT1. Co-expression of IFPN-AKT1 and IFPC-ACTN4 resulted in fluorescence. In contrast, cells with co-expression of IFPN-AKT1 with IFPC only or IFPC-ACTN4Δ310-911 (C terminal truncation of ACTN4) were not fluorescent confirming lack of spontaneous association of the IFP fragments [12]. The reconstitution of IFP fluorescence, representing the formation of a stabilized PDK1-IFPC::IFPN-AKT1 complex, allowed direct observation of the subcellular localization of the complex. In serum-starved cells, fluorescence was observed in the cytoplasm and the cell leading edge (Fig. 1C). The majority of the fluorescence translocated to the cell membrane upon serum stimulation for 10 minutes. Treatment with the PI3K inhibitor LY294002 did not abrogate the formation of the PDK1-IFPC::IFPN-AKT1 complex, as the cells retained fluorescence. However, both basal- and serum-stimulated location of the PDK1-IFPC::IFPN-AKT1 complex to the cell leading edge and the membrane, respectively, were abrogated by LY294002 treatment (Fig. 1C). The localization dynamics of the complex is consistent with that proposed for endogenous AKT and PDK1 [6], [19]. Similar observations regarding PCA-stabilized protein complex subcellular localization following PI3K inhibition have also been previously reported [15]. Thus our results indicate that the subcellular localization but not the formation of the PDK1-IFPC::IFPN-AKT1 complex is dependent on PI3K activity.

Both endogenous PDK1 and AKT have been reported to accumulate in the nucleus following treatment with Leptomycin B (LMB) to inhibit nuclear export [20], [21]. Accumulation of fluorescence in the nucleus was observed in the presence of LMB indicating that the PDK1-IFPC::IFPN-AKT1 complex, similar to the endogenous proteins [6], [21], can translocate into the nucleus (Fig. 1D). LMB treatment did not affect the fluorescence localization at the cell leading edge in serum-starved cells (data not shown). The fluorescence at the cell leading edge was not displayed in Figure 1D because the cell edges were out of focus when the nuclei were in the focal plane. LMB dependent nuclear localization also occurred in the presence of LY294002 (data not shown). Taken together, fusing the complementary fragments of IFP to PDK1 and AKT1 reconstituted IFP fluorescence and facilitated the formation of a PDK1-IFPC::IFPN-AKT1 complex with appropriate subcellular localization.

### PI3K-independent AKT1 phosphorylation and activation by PDK1

**Figure 2:**
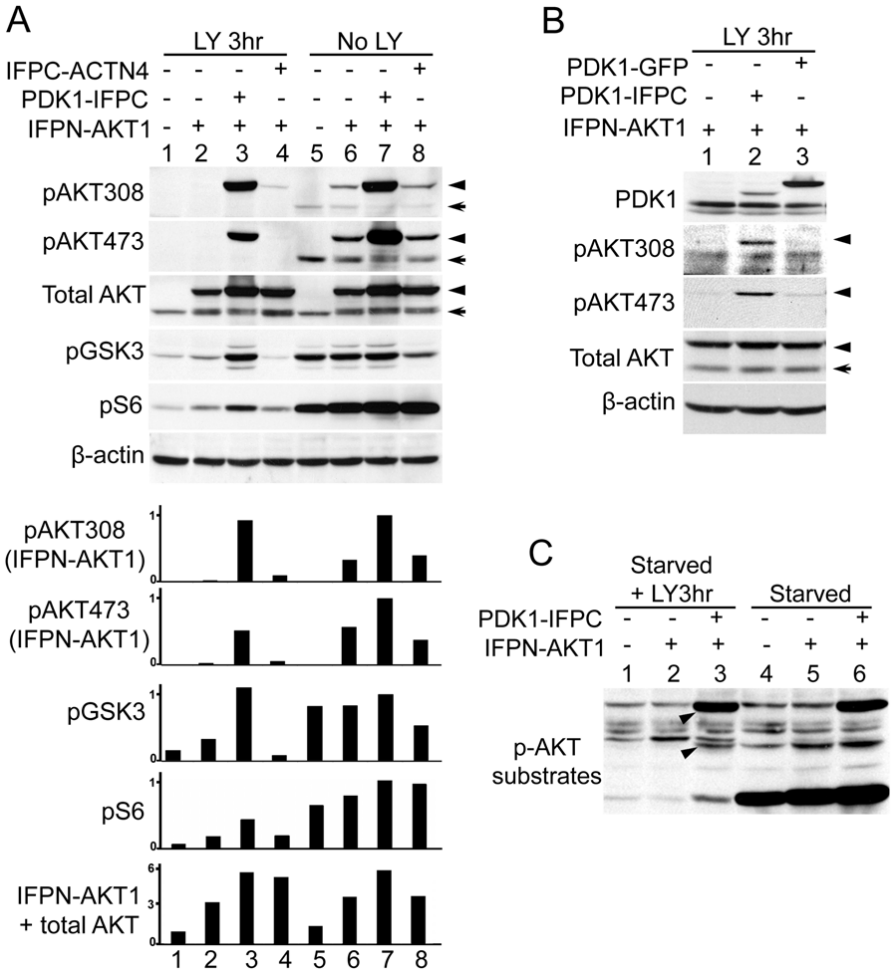
PI3K-independent AKT phosphorylation and activation. (A) AKT phosphorylation and activation in the PDK1-IFPC::IFPN-AKT1 complex. Four stable cell lines, parental HeLa, cells expressing IFPN-AKT1 only, cells co-expressing IFPN-AKT1 and PDK1-IFPC, and cells co-expressing IFPN-AKT1 and IFPC-ACTN4, were serum starved for overnight then treated or not treated with LY294002 (20 µM) for 3 hours. Cells were lysed in RIPA buffer supplied with protease inhibitors and phosphatase inhibitors. Lysates (50 µg/lane) were resolved in 10% SDS PAGE. Antibodies for each blot were listed to the left of the blots. β-actin immunoblotting shows equivalent loading. The arrow head designates IFPN-AKT1. The arrow designates endogenous AKT. Scanning densitometric values of western blots were obtained using the NIH image 1.63.1 software. IFPN-AKT1 phosphorylation was normalized to total IFPN-AKT1. GSK3 (S21/9) and S6 (S235/236) phosphorylation was normalized to β-actin. Data were presented as relative conversion to values of the sample in lane 7. (B) The reconstituted IFP is indispensable for the PI3K-independent IFPN-AKT1 phosphorylation. HeLa cells stably expressing IFPN-AKT1 were transiently transfected with PDK1-IFPC or PDK1-GFP. Cells were serum starved for overnight and then treated with LY294002 (20 µM) for 3 hours. The arrow head designates IFPN-AKT1. The arrow designates endogenous AKT. (C) Phosphorylation of AKT substrates by the PDK1-IFPC::IFPN-AKT1 complex. Parental HeLa, cells stably expressing IFPN-AKT1 only, and cells stably co-expressing IFPN-AKT1 and PDK1-IFPC were serum starved for overnight then treated or not treated with LY294002 (20 µM) for 3 hours. Anti-phospho-AKT substrate antibody was used to detect the phosphorylation of AKT substrates. Two arrow heads designate the protein species with elevated phosphorylation level in the LY294002 treated cells stably expressing the PDK1-IFPC::IFPN-AKT1 complex.

Since IFP reconstitution led to the stabilization of the PDK1-IFPC::IFPN-AKT1 complex with appropriate subcellular localization, we sought to determine whether such stable AKT1 association with PDK1 was sufficient for AKT1 phosphorylation and activation. In HeLa cells expressing IFPN-AKT1 only (Fig. 2A, lane 6), the relative levels of IFPN-AKT1 phosphorylation at T308 and S473 (arrow head) were comparable to that of endogenous AKT (arrows), indicating IFPN-AKT1 was phosphorylated similarly to endogenous AKT. Co-expression with PDK1-IFPC (Fig. 2A, lane 7) led to a substantial increase in IFPN-AKT1 phosphorylation at both sites. Strikingly, while the phosphorylation of AKT in either non-transfected or IFPN-AKT1 alone transfected cells was completed abolished by treatment with LY294002 (Fig. 2A, lane 1 and lane 2), IFPN-AKT1 phosphorylation on T308 in cells co-expressing PDK1-IFPC was resistant to LY294002 treatment (Fig. 2A, lane 3), indicating that PDK1-IFPC phosphorylated IFPN-AKT1 in the complex independent of PI3K activity. In these cells, IFPN-AKT1 was also strongly phosphorylated at S473 in the presence or absence of LY294002, though the phosphorylation was partially inhibited by LY294002, indicating the complex formation stabilized by the reconstituted IFP also promoted AKT1 S473 phosphorylation (Fig. 2A, lane 3 and lane 7). We demonstrated the PI3K-independent IFPN-AKT1 phosphorylation at S473 in the complex is still dependent on the mTOR-rictor complex (mTORC2) by siRNA knock down of rictor [5] (Figure S1). Endogenous AKT phosphorylation remained sensitive to LY294002 in cells co-expressing IFPN-AKT1 and PDK1-IFPC indicative of efficient inhibition of PI3K. To confirm that the phosphorylation of IFPN-AKT1 was dependent on the formation of the PDK1-IFPC::IFPN-AKT1 complex rather than simply due to PDK1 overexpression, we compared IFPN-AKT1 phosphorylation in the cells transiently transfected with PDK1-IFPC or PDK1-GFP. Although the expression level of PDK1-GFP was much higher than PDK1-IFPC, in the presence of LY294002, IFPN-AKT1 was only phosphorylated when co-transfected with PDK1-IFPC (Fig. 2B, lane 2). Thus, PDK1 overexpression without stabilization of the interaction between PDK1 and AKT1 is not sufficient for PI3K-independent AKT phosphorylation. The amount of both transfected AKT1 and PDK1, which was 3–6 fold and 4–8 fold greater than endogenous AKT and PDK1 in HeLa cells, respectively (Fig. 2A and Figure S2), was comparable to the levels found in other cancer cells with amplification of AKT and PDK1 (Figure S3).

The known AKT substrate, GSK3, was phosphorylated at comparable levels in cells expressing the complex with or without LY294002 treatment (Fig. 2A, lane 3 and lane 7), indicating that IFPN-AKT1 in the complex is active and able to efficiently phosphorylate downstream substrates in the presence of LY294002. PDK1 overexpression alone was not sufficient to induce GSK3 phosphorylation (Figure S2). S6 phosphorylation was markedly inhibited by LY294002 treatment compatible with the expected inhibition of mTORC1 by LY294002 (Fig. 2A). In the presence of LY294002, the PDK1-IFPC::IFPN-AKT1 complex partially restored S6 phosphorylation indicating that the complex promotes mTORC1-dependent p70S6K activity (Fig. 2A). To examine the effects of the PDK1-IFPC::IFPN-AKT1 complex on the ability of AKT to phosphorylate multiple substrates, western blotting analysis was performed using an antibody specific for the phosphorylated consensus motif in AKT substrates (Fig. 2C). The presence of the PDK1-IFPC::IFPN-AKT1 complex correlated with marked phosphorylation of several protein species in the presence or absence of LY294002 indicating the PDK1-IFPC::IFPN-AKT1 complex phosphorylates multiple substrates independent of PtdInsP3 (Fig. 2C, arrow heads). However, some substrates were more sensitive to the effects of LY294002 such as the low molecular weight band in Figure 2C. Differential sensitivity of AKT substrates to modification of AKT phosphorylation has been reported previously [22] and may thus reflect the relative sensitivity of S473 phosphorylation to LY294002 in cells expressing the PDK1-IFPC::IFPN-AKT1 complex.

We further confirmed that AKT1 phosphorylation in the PDK1-IFPC::IFPN-AKT1 complex was independent of PI3K activity using the PI3K inhibitor, GDC0941. Similar to LY294002 treatment, AKT1 T308 phosphorylation and GSK3 phosphorylation were comparable with/without GDC0941 treatment. AKT1 phosphorylation at S473 was partially resistant to GDC0941 treatment. S6 phosphorylation was sensitive compatible with GDC0941 also inhibiting mTORC1 activity at the concentrations used (Figure S4).

### The ability of the PH domains of AKT1 and PDK1 to interact with membrane phosphatidylinositols is not required for IFPN-AKT1 T308 phosphorylation in the complex

**Figure 3:**
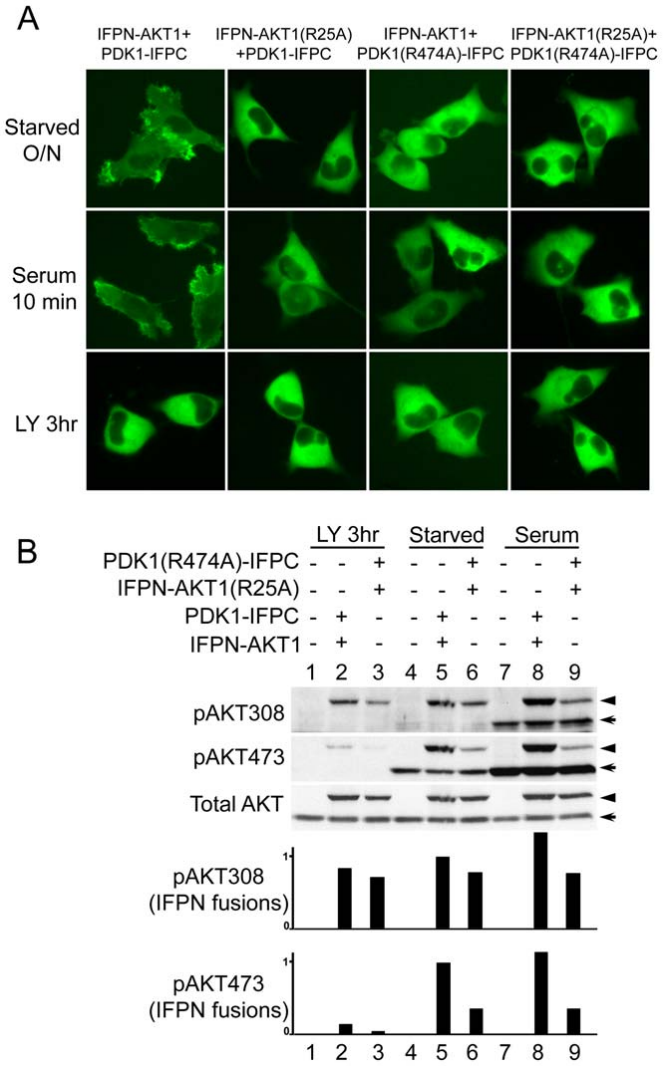
The effects of AKT and PDK1 PH domain mutations on PI3K-independent AKT phosphorylation. (A) PH domain mutations abolish membrane translocation of the PCA-stabilized protein complexes. HeLa cells were transiently transfected to co-express a pair of fusion proteins shown above each column of images. Cells were starved overnight (upper panel) and treated with serum for 10 minutes (middle panel) or LY294002 for 3 hours (lower panel). At least 100 cells were examined from different fields for each sample with above 95% of the examined cells in each sample showing the same fluorescence localization presented. (B) IFPN-AKT1 phosphorylation in the complex is independent of PtdInsP3 binding of both AKT and PDK1. U2OS cells were used because of higher transfection efficiency than HeLa. Cells were transiently co-transfected with IFPN-AKT1 and PDK1-IFPC (lane 2, 5, and 8) or with IFPN-AKT1(R25A) and PDK1(R474A)-IFPC (lane 3, 6, and 9). Cells were serum starved for overnight and then treated with LY294002 (20 µM) or serum (10%) for 3 hours. Parental U2OS was included as controls (lane 1, 4, and 7). Scanning densitometric values of western blots were obtained using the NIH image 1.63.1 software. Phosphorylation of IFPN fusions was normalized to total IFPN fusion proteins. Data were presented as relative conversion to values of the sample in lane 5. The arrow head designates IFPN-AKT1. The arrow designates endogenous AKT.

The PH domains of AKT and PDK1 mediate membrane recruitment through binding to phosphatidylinositol lipids [6], [19]. To confirm that phosphorylation of IFPN-AKT1 by PDK1-IFPC in the complex is independent of PI3K and membrane localization, we created IFPN-AKT1(R25A) and PDK1(R474A)-IFPC PH domain mutants which abrogate membrane localization of each protein [19], [23]. HeLa cells transiently expressing the wild type PDK1-IFPC::IFPN-AKT1 complex (Fig. 3A) displayed the same localization pattern as was seen with stable transfection of the complex in HeLa cells (Fig. 1C) with serum starvation, serum stimulation, and treatment with LY294002. Co-transfection of IFPN-AKT1(R25A) and PDK1(R474A)-IFPC, with the other mutated protein or with the wild-type version of the complementary protein, also resulted in fluorescence, indicating that these mutant proteins formed a stabilized complex in living cells (Fig. 3A). The fluorescent complexes containing either mutant protein, however, failed to demonstrate localization at the cell leading edge or serum-stimulated membrane translocation. Thus, the integrity of PH domains in both AKT1 and PDK1 is required for the membrane localization of the PDK1-IFPC::IFPN-AKT1 complex.

IFPN-AKT1(R25A) was phosphorylated at T308 in the PDK1(R474A)-IFPC::IFPN-AKT1(R25A) complex to an extent comparable to wild type IFPN-AKT1 in the complex, though with a slight decrease, in starved cells with or without LY294002 treatment (Fig. 3B, compare lane 3 with lane 2, or lane 6 with lane 5; relative phosphorylation levels shown in the densitometry bar graphs), further confirming that AKT can be phosphorylated in the complex without membrane localization of AKT and PDK1. In contrast, IFPN-AKT1(R25A) phosphorylation at S473 was reduced comparing with wild type IFPN-AKT1 under starvation conditions with or without LY294002 (Fig. 3B, compare lane 3 with lane 2, or lane 6 with lane 5), indicating that the S473 phosphorylation in the complex demonstrates greater PI3K and membrane localization dependence than the T308 phosphorylation. As expected, the phosphorylation level of IFPN-AKT1(R25A) at T308 and S473 was not increased by serum stimulation (Fig. 3B, compare lane 9 with lane 6), which is consistent with previous work [23].

We further confirmed the PH domain independent phosphorylation of AKT1 in the complex by treatment with a selective AKT PH domain inhibitor, Akti-1/2. (Bilodeau, 2008). As expected, AKT1 T308 phosphorylation and GSK3 phosphorylation were comparable in the presence or absence of Akti-1/2. AKT1 phosphorylation at S473 was partially resistant to the treatment. S6 phosphorylation was unaltered in the presence or absence of Akti-1/2 indicating that the inhibitor has no off target effect on mTORC1 and p70S6K, and the PDK1-IFPC::IFPN-AKT1 complex is responsible for mTORC1-dependent p70S6K activation in the presence of Akti-1/2 (Figure S5).

### The PDK1-IFPC::IFPN-AKT1 complex provides a cell-based platform to examine specificity of drugs targeting PI3K pathway components

**Figure 4:**
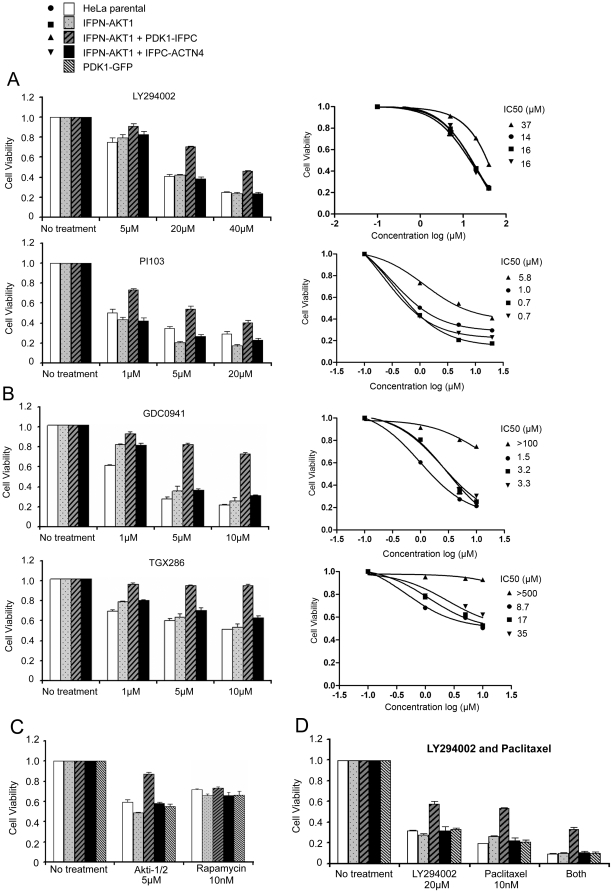
The effects of the PDK1-IFPC::IFPN-AKT1 complex on cell viability. Five cell lines were used in cell viability assays including parental HeLa and four stable HeLa cell lines expressing IFPN-AKT1 alone, co-expressing IFPN-AKT1 and PDK1-IFPC, co-expressing IFPN-AKT1 and IFPC-ACTN4, or expressing PDK1-GFP alone, respectively. Cells were plated in 96-well plates in complete medium at 3000 cells/well. After 18–24 hours, cells were treated with drugs at different concentrations for 24 hours. Cell viability was measured by CellTiter Blue Cell Viability assay according to manufacturer's instructions. Data presented as survival rates normalized to non-treatment controls, respectively. Error bars were the standard deviations of triplicates of each treatment. Cell viability in each treatment was also plotted against the logarithm of drug concentration by fitting to the 3-parameter log(inhibitor) vs. response curve in GraphPad Prism version 5.0. IC50 was calculated by interpolating corresponding cell viability curve. (A) The PDK1-IFPC::IFPN-AKT1 complex promotes cell viability with LY294002 or PI103 treatment. (B) The PDK1-IFPC::IFPN-AKT1 complex promotes cell viability with GDC0941 or TGX286 treatment. (C) The PDK1-IFPC::IFPN-AKT1 complex promotes cell viability with Akti-1/2 but has no effect on cell viability with rapamycin treatment. (D) The PDK1-IFPC::IFPN-AKT1 complex promotes cell viability with paclitaxel treatment and combined treatment with LY294002 and paclitaxel.

The PDK1-IFPC::IFPN-AKT1 complex remained active and did not localize to the membrane in the presence of LY294002, which offered the opportunity to evaluate localization and activity dependent functions of AKT1 and PDK1 separately. Further, the constitutive activation of the PDK1-IFPC::IFPN-AKT1 complex in the presence of LY294002 allowed us to access its specific function in the context of PI3K inhibition, which abrogates endogenous signaling. We thus determined whether the PDK1-IFPC::IFPN-AKT1 complex could promote cell viability, a major cellular function of the endogenous PDK1 and AKT, when particular PI3K pathway components were inhibited. The expression of the PDK1-IFPC::IFPN-AKT1 complex, but not IFPN-AKT1 either alone or with IFPC-ACTN4, promoted cell viability in the presence of PI3K inhibitors, LY294002 and PI103, indicating the hyperactive PDK1-IFPC::IFPN-AKT1 was sufficient to increase cell viability in the absence of PI3K signaling (Fig. 4A). The effects were dependent on formation of the PDK1-IFPC::IFPN-AKT1 complex as IFPN-AKT1:IFPC-ACTN4 or PDK1-GFP alone was not sufficient to increase viability in the presence of LY294002 or PI103 (Fig. 4A and D). The complex only partially bypassed the effects of LY294002 or PI103. As both LY294002 and PI103 inhibit other targets including mTOR, this could indicate off target effects of these compounds such as inhibition of mTOR-p70S6K in cell viability[24]. We also tested two other PI3K inhibitors that are proposed to have less activity on mTOR, GDC0941 and TGX286 (Fig. 4B). GDC0941 is a pan PI3K inhibitor with weak inhibition on mTORC1[25]. The PDK1-IFPC::IFPN-AKT1 complex promotes cell viability in the presence of GDC0941 (Fig. 4B). Further, while GDC0941 and LY294002 suppressed cell viability to a similar degree in control cells, i.e. GDC0941 at 5 µM and LY294002 at 20 µM, the PDK1-IFPC::IFPN-AKT1 complex restored cell viability to a significantly higher level when treated with GDC0941 than treated with LY294002 or PI103 (Fig. 4A and B), consistent with GDC0941 has less off target activity than LY294002 and PI103. TGX286 is a selective inhibitor for PI3Kβ/δ without inhibition on mTOR complexes (Knight 2006). The PDK1-IFPC::IFPN-AKT1 complex completely restores cell viability with TGX286 treatment (Fig. 4B), indicating the drug has no or little off target effects other than inhibiting PI3K, which could be completely bypassed by the PDK1-IFPC::IFPN-AKT1 complex. The data also suggest that AKT1 is the primary mediator downstream of the drug targets, PI3Kβ/δ, in promoting cell viability. Therefore, this approach could potentially distinguish between on and off target activity of PI3K inhibitors in functional outcomes. It is important to note that the current studies were performed with AKT1, thus effects of AKT2 and AKT3 would be inhibited by LY294002, PI103, or GDC0941. So the PDK1-IFPC::IFPN-AKT1 complex provides the opportunity to explore the effects of selective activation of AKT1 in the absence of activation of AKT2 or AKT3.

The PDK1-IFPC::IFPN-AKT1 complex also reduced sensitivity to the selective AKT1 and AKT2 PH domain inhibitor, Akti-1/2 [26], [27] (Fig. 4C), confirming the activation of IFPN-AKT1 in the complex is PH domain independent as shown in Figure 3B and Figure S5. Once again there was only partial protection compatible with off target effects of the inhibitor or effects on AKT2. In contrast, the PDK1-IFPC::IFPN-AKT1 did not render cells resistant to rapamycin, an inhibitor of mTOR, which is downstream of AKT (Fig. 4C), confirming that mTORC1 is the primary mediator downstream of AKT1 in promoting cell viability. Rapamycin reduced the cell viability by ∼30% at the concentration of 10 nM (Fig. 4C). Higher concentration of rapamycin (up to 1 µM) did not further reduce the cell viability (Figure S6), compatible with 10 nM of rapamycin completely inhibiting mTORC1 and higher concentration of rapamycin not exhibiting additional off target effects on cell viability. The data indicated that complete mTORC1 inhibition would induce an approximately ∼30% reduction in cell viability in these cells. PI103 is a dual PI3K and mTORC1 inhibitor[24]. PI103 treatment at 1 µM reduced cell viability by ∼30% in cells expressing the PDK1-IFPC::IFPN-AKT1 complex (Fig. 4A), which is consistent with the ∼30% reduction in cell viability with rapamycin treatment (Fig. 4C). In contrast, PI103 treatment at 1 µM reduced cell viability by ∼60% in control cells (Fig. 4A), suggesting in addition to the ∼30% reduction induced by mTORC1 inhibition, there was another ∼30% reduction induced by PI3K inhibition in control cells. The data suggest that the PDK1-IFPC::IFPN-AKT1 complex, completely bypassed the ∼30% reduction in viability induced by PI3K inhibition but retained sensitivity to the ∼30% reduction in viability induced by mTORC1 inhibition. The data also imply that the effects of PI3K inhibition and mTORC1inhibition are additive. Further, the data suggest that PI103 has no or little effects other than inhibiting PI3K and mTORC1 on cell viability, because PI3K inhibition was completely bypassed by the PDK1-IFPC::IFPN-AKT1 complex. LY294002 treatment at 20 µM exhibited similar inhibition pattern, ∼60% of reduction in control cells and ∼30% of reduction in cells expressing the PDK1-IFPC::IFPN-AKT1 complex. However, LY294002 and PI103 at higher concentrations may exhibit additional off target effects. Although PI103 and LY294002 have been shown to inhibit other kinases including DNA-PK, ATR[24], the data suggest that the inhibition of PI3K and mTORC1 primarily contributes to cell viability reduction.

Six drugs targeting the components in the PI3K-AKT-mTor signaling were tested (Table 1). The PDK1-IFPC::IFPN-AKT1 complex was resistant to inhibition of PI3K upstream of AKT, as well as inhibition of the AKT PH domain, while retaining sensitivity to inhibitors that target the pathway downstream of AKT. The PDK1-IFPC::IFPN-AKT1 complex was only partially resistant to LY294002 and PI103 likely due to the ability of the two drugs to effectively inhibit mTORC1 as well as PI3K. The PDK1-IFPC::IFPN-AKT1 complex exhibited partial resistance to GDC0941 likely because GDC0941 inhibits mTORC1 though weaker than LY294002 and PI103. The PDK1-IFPC::IFPN-AKT1 complex exhibited complete resistance to TGX286, a selective PI3Kβ/δ inhibitor without inhibition on mTORC1.

**Table 1:** Drug sensitivity in cells expressing the PDK1-IFPC::IFPN-AKT1 complex.

No.	Drugs	Known targets	Drug sensitivity in cells expressing the PDK1-IFPC::IFPN-AKT1 complex*
1	LY294002	Pan PI3K, mTORC1	+
2	PI103	Pan PI3K, mTORC1	+
3	GDC0941	Pan PI3K, mTORC1	++
4	TGX286	PI3Kβ/δ	+++
5	Akti-1/2	AKT1, AKT2	++
6	Rapamycin	mTORC1	−

*“+++” indicates completely resistant. “−” indicates completely sensitive.

The PDK1-IFPC::IFPN-AKT1 complex could also be used to examine if AKT1 activation is responsible for a resistance to drugs targeting non PI3K pathway mediated events. As shown in Figure 4D, the PDK1-IFPC::IFPN-AKT1 complex mediated partial resistance to paclitaxel and to combined treatment with LY294002 and paclitaxel (Fig. 4D and Figure S6), indicating that AKT1 contributes to drug resistance to paclitaxel in the cells examined. Cells treated with both LY294002 and paclitaxel exhibited lower cell viability than treated with the individual drug indicating paclitaxel inhibits cell viability, at least partially, independent of PI3K-AKT-mTor signaling.

Our results suggest that mechanisms independent of membrane PtdIns could enable AKT phosphorylation by PDK1. For example, any protein or other molecules that enhances PDK1::AKT interaction or alters conformation of the complex could be sufficient to promote AKT phosphorylation and activation. Recently, p21-activated protein kinase 1 (PAK1) was shown to serve as a scaffold to promote AKT interaction with PDK1 and subsequently AKT phosphorylation [9], which is consistent with this contention. Indeed a PDK1 PH domain mutant (K465E) which lacks PtdInsP3 binding, retained an ability to phosphorylate AKT in knock-in mice, albeit at a reduced level [28]. Furthermore, stress-induced phosphorylation of AKT at T308 can be PI3K independent, while S473 phosphorylation is still PI3K dependent [29], [30], [31]. The stress-induced AKT activation may result from enhanced PDK1::AKT interaction independent of PtdInsP3 and membrane localization. PI3K-independent AKT activation may play an important role in promoting cancer cell survival since cancer cells usually exist in stressed physiological conditions lacking sufficient growth factors, nutrients, or oxygen.

Recently, AKT has been shown to form a complex with PDK1 in resting cells but the intramolecular interaction between AKT PH domain and kinase domain prevents the phosphorylation by PDK1. PtdInsP3 binding induces a conformational change enabling T308 phosphorylation (Calleja, 2007). The PDK1-IFPC::IFPN-AKT1 complex in our study represents a potent association between AKT1 and PDK1 which is sufficient for T308 phosphorylation on AKT1. Thus we postulate that, besides PtdInsP3, other molecules could also induce functional associations between PDK1 and AKT. Our results suggest that direct targeting of the PDK1::AKT interaction is a possible strategy for cancer drug development.

In summary, we have employed PCA as a novel tool to characterize AKT1 phosphorylation by PDK1 and provided direct evidence showing stabilized AKT1 association with PDK1 by the reconstituted IFP is sufficient for AKT1 phosphorylation by PDK1 independent of PI3K and membrane localization. PtdInsP3 binding and membrane localization are not prerequisites for phosphorylation of T308 in AKT by PDK1, but rather appear to function to induce association of PDK1 and AKT in a conformation that allows AKT phosphorylation. The reconstituted IFP functions to associate AKT1 and PDK1, similar to the function of PtdInsP3 in normal cells. The complex generated in the study proved to be useful to test different inhibitors and dissect specific functions including off target effects of drugs. The same procedure could be used to characterize other protein complexes.

## Materials and Methods

### Cell culture and plasmids

HeLa cell line used in the study is a Tet-on derivative from BD Clontech (Palo Alto, CA). U2OS was from ATCC (Manassas, VA). Cells were cultured in Dulbecco's modified Eagle's medium (Invitrogen, Carlsbad, CA) supplemented with 10% fetal calf serum. Plasmids 11117-Y101 (expressing IFPN-AKT1) and 21622-Y108 (expressing PDK1-IFPC) were from Odyssey Thera. Inc. (San Ramon, CA) [15]. A plasmid expressing PDK1-GFP was constructed by replacing the IFPC fragment on 21622-Y108 with full length GFP. Plasmids expressing the two PH domain mutants, IFPN-AKT1(R25A) and PDK1(R474A)-IFPC, were constructed by mutagenesis from 11117-Y101 and 21622-Y108 with Quikchange mutagenesis kit (Stratagene, La Jolla, CA) and confirmation by sequencing. Construction of IFPC-ACTN4Δ310-911 was described earlier[12]. The cells were transfected using FuGENE 6 transfection reagent (Roche, Indianapolis, IN) following the manufacturer's protocol.

### Reagents and antibodies

LY294002 was from CalBiochem (San Diego, CA). PI103 was synthesized at M. D. Anderson Cancer Center according to the structure reported [24]. GDC0941 was from Axon (Groningen, The Netherlands). TGX286 was from GSK (Research Triangle Park, NC). AKT1/2 PH domain inhibitor, Akti-1/2, (1,3-dihydro-1-(1-((4-(6-phenyl-1H-imidazo[4,5-g]quinoxalin-7-yl)phenyl)methyl)-4-piperidinyl)-2H-benzimidazol-2-one) was synthesized (Patent Number WO2003086404) by Keith Woods at Abbott Laboratories (Abbott Park, IL). Rapamycin was from Cell Signaling Technology, Inc. (Beverly, MA). Paclitaxel was from Bristol-Myers Squibb (Princeton, NJ). Leptomycin B (LMB) was from Sigma (St. Louis, MO). Goat AKT1 antibody and mouse PDK1 antibody for Co-IP, normal serum, protein A/G agarose, anti-total GSK3, and β-actin antibody were from Santa Cruz Biotechnology, Inc. (Santa Cruz, CA). Western blotting antibodies for total AKT, phospho-AKT, phospho-AKT substrates, phospho-GSK3, phospho-S6, total PDK1, and Rictor were from Cell Signaling Technology, Inc.

### RNAi, co-immunoprecipitation (Co-IP), and western blotting

Four siRNAs targeting human Rictor and a non-targeting siRNA pool were from Dharmacon (Lafayette, CO). RNAi silencing was performed according to manufacturer's protocol. To prepare cell lysates for western blotting, cells were lysed in RIPA buffer (Tris-HCl, pH 7.4, 50 mM; NaCl, 150 mM; NP-40, 1%; Sodium deoxycholate, 0.5%; SDS, 0.1%) supplemented with protease inhibitor and phosphatase inhibitor cocktail (Pierce, Rockford, IL). To prepare cell lysates for Co-IP, cells were lysed in NP40 buffer (Tris-HCl, pH 8.0, 50 mM; NaCl, 150 mM; NP-40, 1%) with protease inhibitor cocktail. Co-IP and western blotting were performed as previously described [32].

### Cell Viability assay

Cells were plated into 96-well plates at 3000 cells/well and incubated for 18–24 hours then treated with drugs for 24 hours. Cell viability was determined by CellTiter Blue Cell Viability assay (Promega, Madison, WI) according to manufacturer's protocol.

## Supporting Information

Figure S1 IFPN-AKT1 phosphorylation on S473 in the complex is mTORC2 dependent. HeLa cells stably co-expressing the PDK1-IFPC::IFPN-AKT1 complex were transfected with Rictor siRNA or a non-targeting siRNA (NT-siRNA) pool. Twenty-four hours post-transfection, cells were serum starved for overnight and then treated with LY294002 (20 µM) for 3 hours. Parental HeLa cells transfected with Rictor siRNA or non-targeting siRNA were included as controls (cultured in complete medium). The arrow head designates IFPN-AKT1. The arrow designates endogenous AKT. siRNA-mediated Rictor depletion led to a substantial reduction in IFPN-AKT1 S473 phosphorylation, while T308 phosphorylation was not affected, indicating that S473 phosphorylation was dependent on mTORC2 activity in the complex. GSK3 was comparably phosphorylated with or without Rictor knockdown, suggesting that IFPN-AKT1 phosphorylated at T308 retained the ability to phosphorylate GSK3 despite a substantial decrease in S473 phosphorylation. Multiple independent Rictor siRNA were used with similar results (data not shown).
(0.60 MB TIF)

Figure S2 PDK1 overexpression alone is not sufficient for the phosphorylation of GSK3. Four cell lines, parental HeLa, cells stably expressing IFPN-AKT1 only, cells stably co-expressing IFPN-AKT1 and PDK1-IFPC, and cells stably expressing PDK1-GFP alone were serum starved for overnight then treated with LY294002 (20 µM) for 3 hours. The arrow head designates IFPN-AKT1. The arrow designates endogenous AKT. Phosphorylation of GSK3 was blocked by LY294002 in cells overexpressing PDK1-GFP alone, indicating that PDK1 overexpression was not sufficient for the observed PI3K-independent phosphorylation of AKT substrates.
(0.41 MB TIF)

Figure S3 Comparison of HeLa cell AKT and PDK1 levels with other cancer cell lines. Quantitative Reverse Phase Protein Arrays (RPPA) was performed at the CCSG RPPA core facility at MD Anderson Cancer Center to determine relative levels of AKT and PDK1 in different cell lines. The cell lines, including MCF7(Breast), T47D(Breast), UACC62(Melanoma), M14(Melanoma), MALME(Melanoma), WM3451(Melanoma), HOP-62(Lung), COLO205(Colon), HCC2998(Colon), and K562(Leukemia), were shown for relative AKT or PDK1 level comparing with HeLa. Higher levels of AKT (2–4 fold of HeLa AKT) and PDK1 (5–6 fold of HeLa PDK1) were frequently seen in other cancer cell lines.
(0.13 MB TIF)

Figure S4 AKT phosphorylation and activation in the PDK1-IFPC::IFPN-AKT1 complex with GDC0941 treatment. Four cell lines, parental HeLa, cells stably expressing IFPN-AKT1 only, co-expressing IFPN-AKT1 and PDK1-IFPC, and co-expressing IFPN-AKT1 and IFPC-ACTN4, were serum starved for overnight then treated or not treated with GDC0941 (10 µM) for 3 hours. Cells were lysed in RIPA buffer supplied with protease inhibitors and phosphatase inhibitors. Lysates (50 µg/lane) were resolved in 10% SDS PAGE. Antibodies for each blot were listed to the left of the blots. Beta-actin immunoblotting shows equivalent loading. The arrow head designates IFPN-AKT1. The arrow designates endogenous AKT. Scanning densitometric values of western blots were obtained using the NIH image 1.63.1 software. IFPN-AKT1 phosphorylation was normalized to total IFPN-AKT1. GSK3(S21/9) phosphorylation was normalized to total GSK3. S6(S235/236) phosphorylation was normalized to beta-actin. Data were presented as relative conversion to values of the sample in lane 7.
(1.23 MB TIF)

Figure S5 AKT phosphorylation and activation in the PDK1-IFPC::IFPN-AKT1 complex with Akti-1/2 treatment. The experiments was performed and the data were processed the same as above in Figure S3 except for the cells were treated with Akti-1/2 at 5 µM.
(1.28 MB TIF)

Figure S6 The effects of the PDK1-IFPC::IFPN-AKT1 complex on cell viability. Three cell lines were used in cell viability assays including parental HeLa and two stable HeLa cell lines expressing IFPN-AKT1 alone or co-expressing IFPN-AKT1 and PDK1-IFPC. Cells were plated in 96-well plates in complete medium at 3000 cells/well. After 18–24 hours, cells were treated with drugs at different concentrations for 24 hours. Cell viability was measured by CellTiter Blue Cell Viability assay according to manufacturer's instructions. Data presented as survival rates normalized to non-treatment controls, respectively. Error bars were the standard deviations of triplicates of each treatment. (A) The PDK1-IFPC::IFPN-AKT1 complex has no effect on cell viability with Rapamycin treatment. (B) The PDK1-IFPC::IFPN-AKT1 complex promotes cell viability with paclitaxel treatment.
(1.45 MB TIF)
