# Supplementary figures and images for: Physical Association of PDK1 with AKT1 Is Sufficient for Pathway Activation Independent of Membrane Localization and Phosphatidylinositol 3 Kinase
Source: PLoS One. 2010 Mar 26;5(3):e9910. doi: 10.1371/journal.pone.0009910 (PMC2845649; doi:10.1371/journal.pone.0009910)

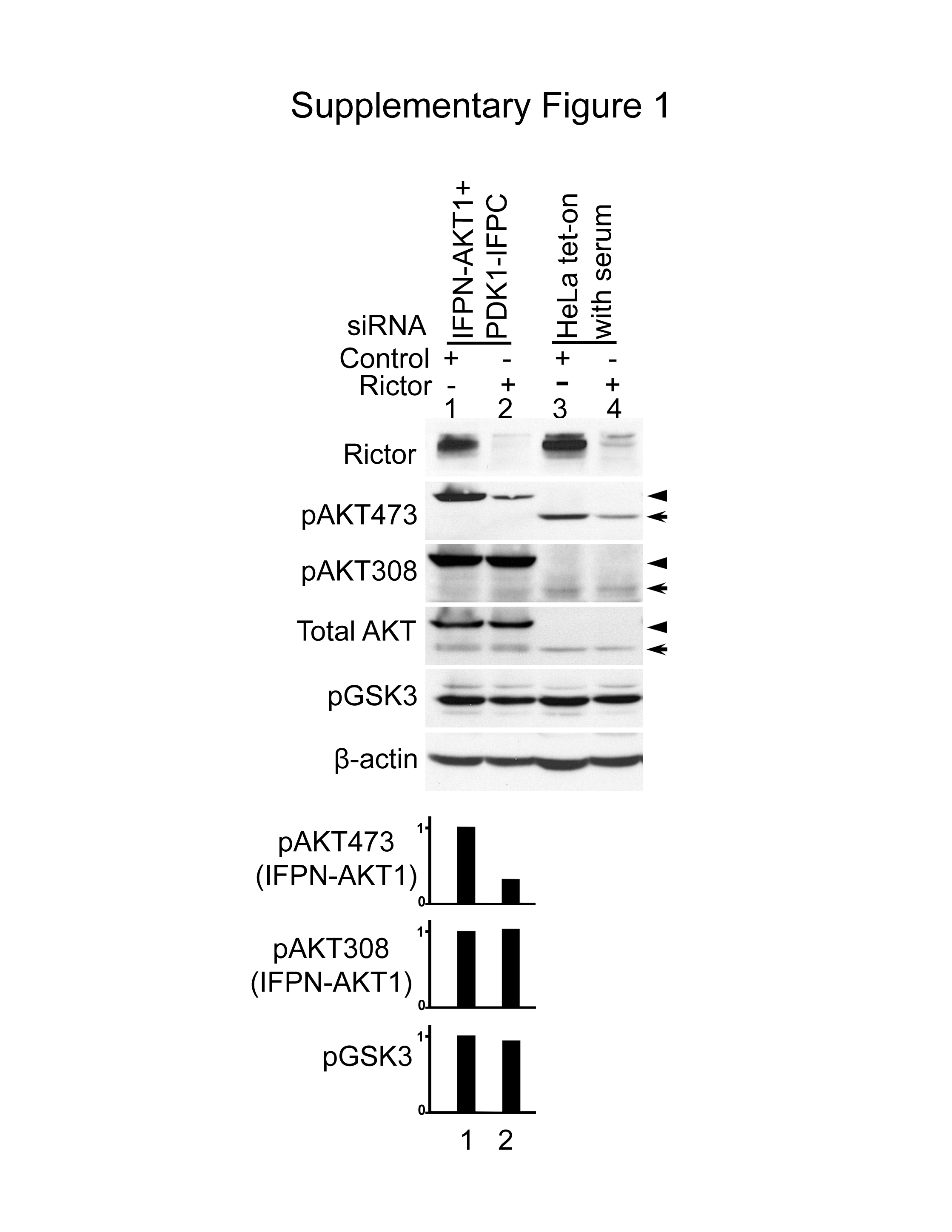

Supplement: Figure S1 — IFPN-AKT1 phosphorylation on S473 in the complex is mTORC2 dependent. HeLa cells stably co-expressing the PDK1-IFPC::IFPN-AKT1 complex were transfected with Rictor siRNA or a non-targeting siRNA (NT-siRNA) pool. Twenty-four hours post-transfection, cells were serum starved for overnight and then treated with LY294002 (20 µM) for 3 hours. Parental HeLa cells transfected with Rictor siRNA or non-targeting siRNA were included as controls (cultured in complete medium). The arrow head designates IFPN-AKT1. The arrow designates endogenous AKT. siRNA-mediated Rictor depletion led to a substantial reduction in IFPN-AKT1 S473 phosphorylation, while T308 phosphorylation was not affected, indicating that S473 phosphorylation was dependent on mTORC2 activity in the complex. GSK3 was comparably phosphorylated with or without Rictor knockdown, suggesting that IFPN-AKT1 phosphorylated at T308 retained the ability to phosphorylate GSK3 despite a substantial decrease in S473 phosphorylation. Multiple independent Rictor siRNA were used with similar results (data not shown). (0.60 MB TIF) [file pone.0009910.s001.tif]

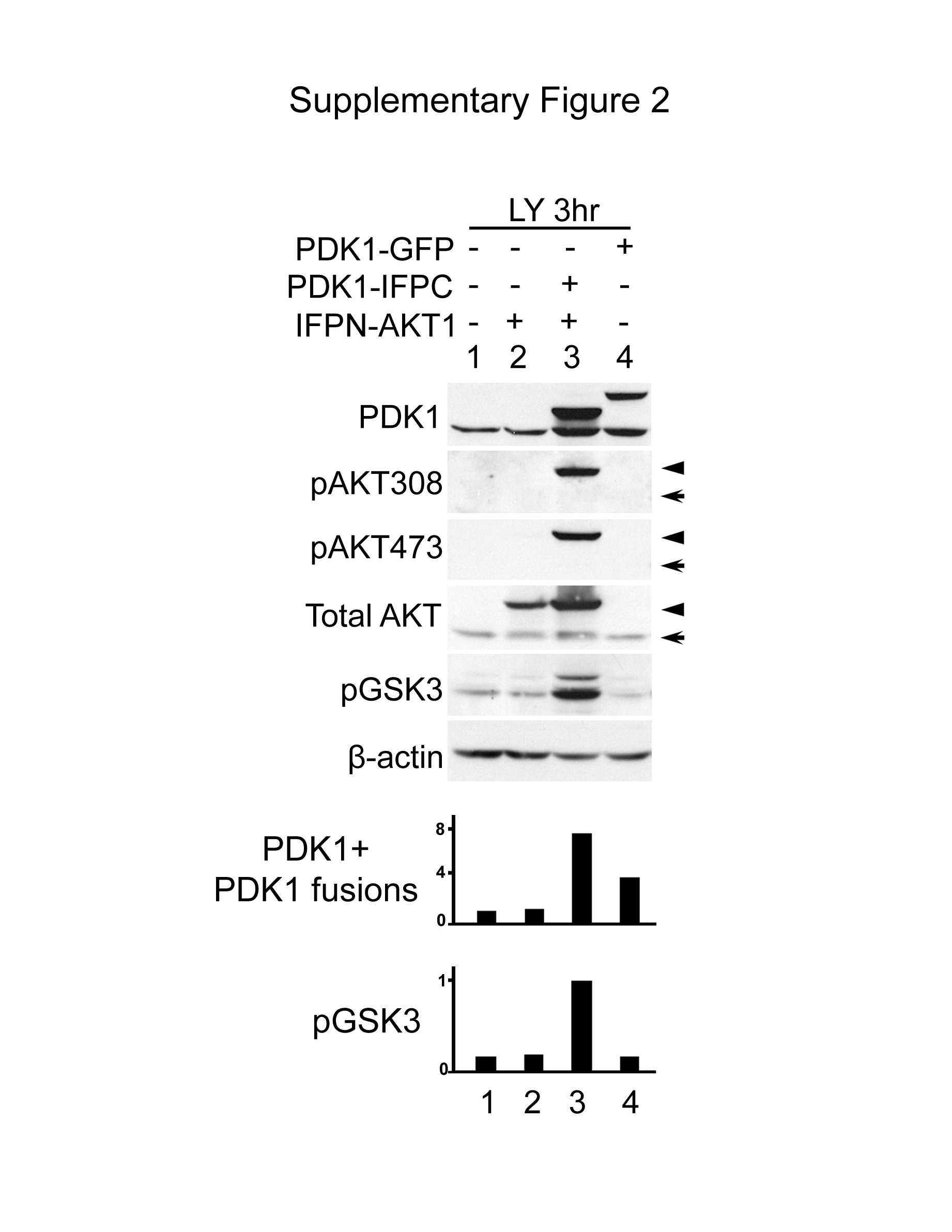

Supplement: Figure S2 — PDK1 overexpression alone is not sufficient for the phosphorylation of GSK3. Four cell lines, parental HeLa, cells stably expressing IFPN-AKT1 only, cells stably co-expressing IFPN-AKT1 and PDK1-IFPC, and cells stably expressing PDK1-GFP alone were serum starved for overnight then treated with LY294002 (20 µM) for 3 hours. The arrow head designates IFPN-AKT1. The arrow designates endogenous AKT. Phosphorylation of GSK3 was blocked by LY294002 in cells overexpressing PDK1-GFP alone, indicating that PDK1 overexpression was not sufficient for the observed PI3K-independent phosphorylation of AKT substrates. (0.41 MB TIF) [file pone.0009910.s002.tif]

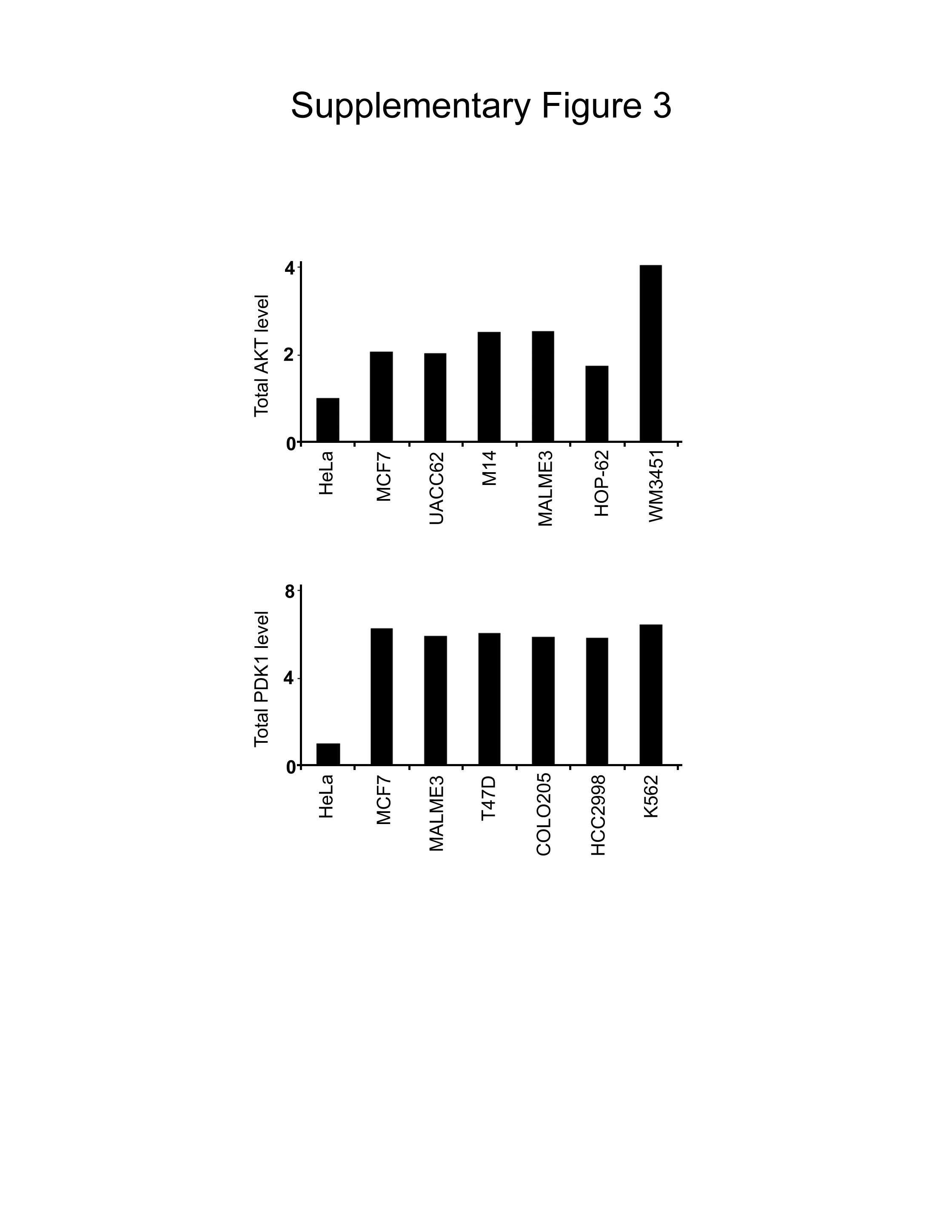

Supplement: Figure S3 — Comparison of HeLa cell AKT and PDK1 levels with other cancer cell lines. Quantitative Reverse Phase Protein Arrays (RPPA) was performed at the CCSG RPPA core facility at MD Anderson Cancer Center to determine relative levels of AKT and PDK1 in different cell lines. The cell lines, including MCF7(Breast), T47D(Breast), UACC62(Melanoma), M14(Melanoma), MALME(Melanoma), WM3451(Melanoma), HOP-62(Lung), COLO205(Colon), HCC2998(Colon), and K562(Leukemia), were shown for relative AKT or PDK1 level comparing with HeLa. Higher levels of AKT (2–4 fold of HeLa AKT) and PDK1 (5–6 fold of HeLa PDK1) were frequently seen in other cancer cell lines. (0.13 MB TIF) [file pone.0009910.s003.tif]

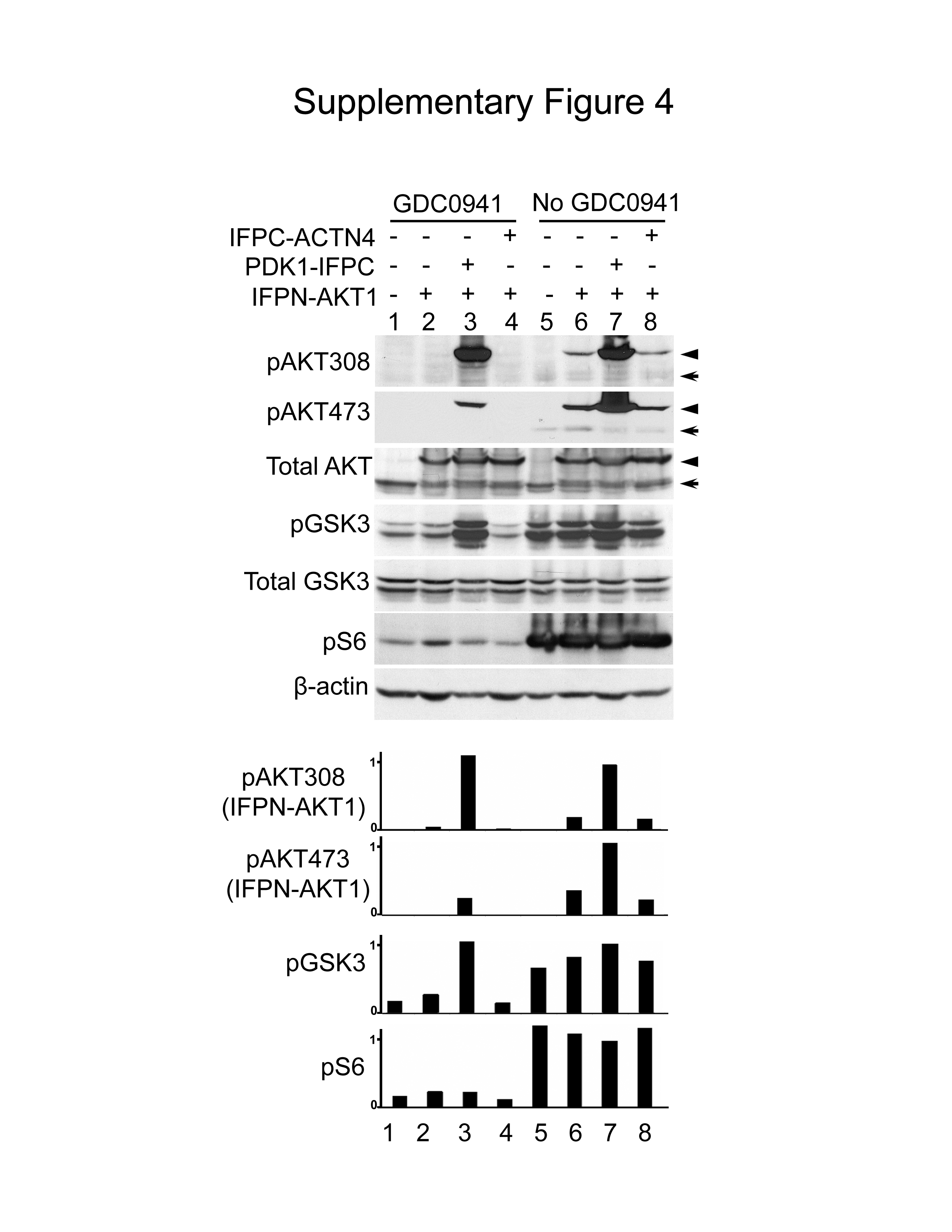

Supplement: Figure S4 — AKT phosphorylation and activation in the PDK1-IFPC::IFPN-AKT1 complex with GDC0941 treatment. Four cell lines, parental HeLa, cells stably expressing IFPN-AKT1 only, co-expressing IFPN-AKT1 and PDK1-IFPC, and co-expressing IFPN-AKT1 and IFPC-ACTN4, were serum starved for overnight then treated or not treated with GDC0941 (10 µM) for 3 hours. Cells were lysed in RIPA buffer supplied with protease inhibitors and phosphatase inhibitors. Lysates (50 µg/lane) were resolved in 10% SDS PAGE. Antibodies for each blot were listed to the left of the blots. Beta-actin immunoblotting shows equivalent loading. The arrow head designates IFPN-AKT1. The arrow designates endogenous AKT. Scanning densitometric values of western blots were obtained using the NIH image 1.63.1 software. IFPN-AKT1 phosphorylation was normalized to total IFPN-AKT1. GSK3(S21/9) phosphorylation was normalized to total GSK3. S6(S235/236) phosphorylation was normalized to beta-actin. Data were presented as relative conversion to values of the sample in lane 7. (1.23 MB TIF) [file pone.0009910.s004.tif]

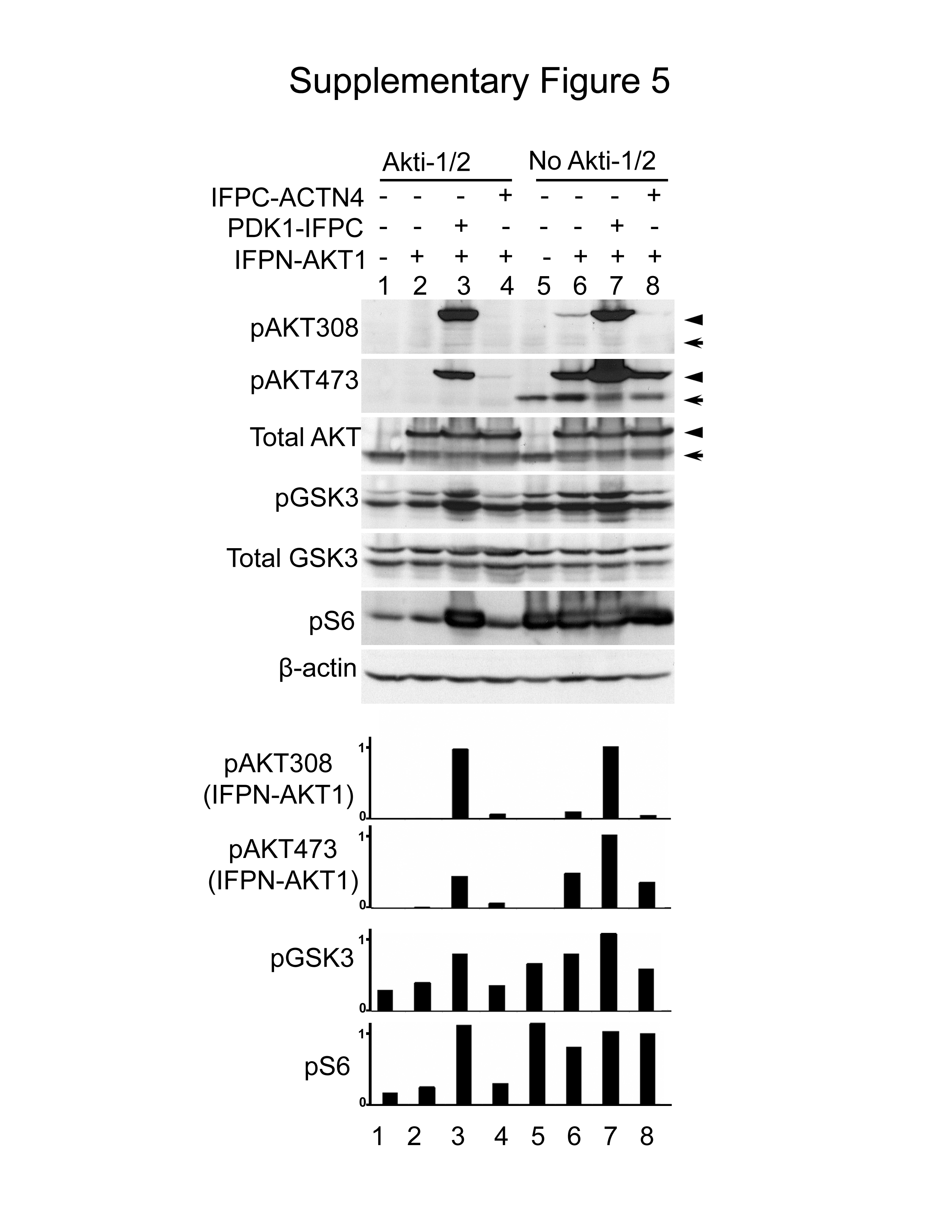

Supplement: Figure S5 — AKT phosphorylation and activation in the PDK1-IFPC::IFPN-AKT1 complex with Akti-1/2 treatment. The experiments was performed and the data were processed the same as above in Figure S3 except for the cells were treated with Akti-1/2 at 5 µM. (1.28 MB TIF) [file pone.0009910.s005.tif]

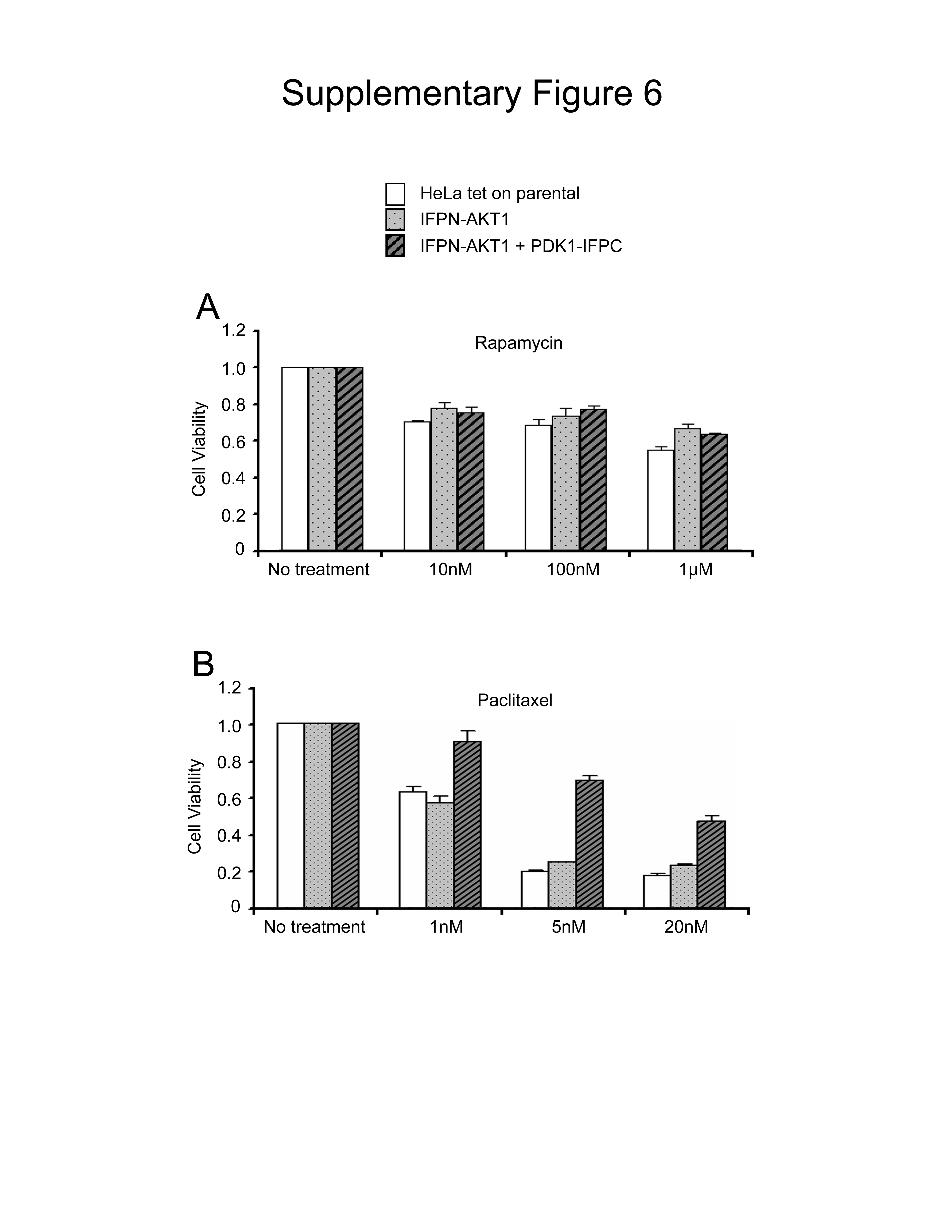

Supplement: Figure S6 — The effects of the PDK1-IFPC::IFPN-AKT1 complex on cell viability. Three cell lines were used in cell viability assays including parental HeLa and two stable HeLa cell lines expressing IFPN-AKT1 alone or co-expressing IFPN-AKT1 and PDK1-IFPC. Cells were plated in 96-well plates in complete medium at 3000 cells/well. After 18–24 hours, cells were treated with drugs at different concentrations for 24 hours. Cell viability was measured by CellTiter Blue Cell Viability assay according to manufacturer's instructions. Data presented as survival rates normalized to non-treatment controls, respectively. Error bars were the standard deviations of triplicates of each treatment. (A) The PDK1-IFPC::IFPN-AKT1 complex has no effect on cell viability with Rapamycin treatment. (B) The PDK1-IFPC::IFPN-AKT1 complex promotes cell viability with paclitaxel treatment. (1.45 MB TIF) [file pone.0009910.s006.tif]
